# Arthroscopic Management of Femoroacetabular Impingement: Current Concepts

**DOI:** 10.3390/jcm14051455

**Published:** 2025-02-21

**Authors:** Filippo Migliorini, Marco Pilone, Ludovico Lucenti, Tommaso Bardazzi, Gennaro Pipino, Raju Vaishya, Nicola Maffulli

**Affiliations:** 1 Department of Life Sciences, Health, and Health Professions, Link Campus University, Via del Casale di San Pio V, 00165 Rome, Italy; 2 Department of Orthopaedic and Trauma Surgery, Academic Hospital of Bolzano (SABES-ASDAA), 39100 Bolzano, Italy; tommaso.bardazzi@sabes.it; 3 Residency Program in Orthopedics and Traumatology, University of Milan, 20133 Milan, Italy; m.pilone97@gmail.com; 4 Department of Precision Medicine in Medical, Surgical and Critical Care (Me.Pre.C.C.), University of Palermo, 90133 Palermo, Italy; ludovico.lucenti@gmail.com; 5 Department of Orthopaedic Surgery, Villa Erbosa Hospital, San Raffaele University, 20132 Milan, Italy; dottgennaropipino@yahoo.it; 6 Department of Orthopaedic and Trauma Surgery, Indraprastha Apollo Hospitals, New Delhi 110076, India; raju.vaishya@gmail.com; 7 Department of Trauma and Orthopaedic Surgery, Faculty of Medicine and Psychology, University La Sapienza, 00185 Rome, Italy; n.maffulli@qmul.ac.uk; 8 School of Pharmacy and Bioengineering, Faculty of Medicine, Keele University, Stoke on Trent ST4 7QB, UK; 9 Centre for Sports and Exercise Medicine, Barts and the London School of Medicine and Dentistry, Mile End Hospital, Queen Mary University of London, London E1 4DG, UK

**Keywords:** femoroacetabular impingement, FAI, arthroscopy, sports

## Abstract

**Background:** Femoroacetabular impingement (FAI) is a common cause of hip pain and dysfunction, especially in young and active individuals, and it may require surgical management for associated labral tears and cartilage damage. The management of FAI has advanced radically over the last few years, and hip arthroscopy has gained a leading role. However, despite the increasing number of published research and technological advancements, a comprehensive systematic review summarising current evidence is still missing. **Methods**: All the clinical studies investigating the arthroscopic management of FAI were accessed. Only studies with a minimum of six months of follow-up were considered. The 2020 PRISMA guidelines were followed. In December 2024, PubMed, Web of Science, and Embase were accessed without time constraints. **Results**: The present systematic review included 258 clinical investigations (57,803 patients). The mean length of follow-up was 34.2 ± 22.7 months. The mean age was 34.7 ± 5.3, and the mean BMI was 25.1 ± 2.0 kg/m^2^. **Conclusions**: The present systematic review updates current evidence on patients who have undergone arthroscopic surgery for FAI, updating and discussing current progress in managing labral injuries and patient selection, emphasising outcomes and pitfalls. Progress in surgery and improvement in eligibility criteria, as well as current controversies and prospects, were also discussed.

## 1. Introduction

In femoroacetabular impingement (FAI), there is irregular contact between the acetabulum and the femoral head [1,2]. FAI is a frequent cause of hip pain and dysfunction, especially in young and active individuals, and it may need surgical treatment for associated labral tears and cartilage damage [3,4]. Two main types of FAI are described: cam impingement, with a not perfectly round femoral head, and pincer impingement, consisting of an acetabulum that overcovers the femoral head [5,6,7]. A mixed-type morphology, including pincer and cam types, is also reported and is the most frequent form [6,8,9]. Many authors have explored several aspects of FAI management, from diagnostic evaluation and surgical techniques to postoperative rehabilitation and long-term outcomes. Some authors advocate non-operative management, while others support the surgical approach to treat the pathology [10,11], with much controversy and unclear guidelines.

The management of FAI has advanced radically over the last few years, and hip arthroscopy has gained a leading role [11,12,13]. Arthroscopic management has gained popularity because it is a mini-invasive procedure that permits a clear vision of the joint and efficient treatment, promising clinical outcomes [14,15,16,17]. The first studies on hip arthroscopy focused mainly on symptomatic relief and functional improvement in the short term [18,19,20]. However, the latest research has outlined favourable long-term outcomes, efficiency across different patient demographics, and efficacy compared to other treatment modalities [21,22]. The increasing success of hip arthroscopy arises from the possibility of undertaking a comprehensive assessment and management of intra-articular pathologies, such as labral tears, cartilage lesions, and bony impingements, with less tissue damage compared to open techniques [23,24,25,26,27]. The number of studies on arthroscopic management of FAI is on the increase [11,28,29,30,31,32,33], but they convey variable and inconsistent information [34,35,36]. An essential lack of standardised international guidelines and consensus on ideal management for FAI is still present [28,37,38,39]. The large variability of patients’ characteristics and types of treatment leads to the necessity of delineating updated and standardised diagnostic and therapeutic approaches that are valid within different contexts [9,40]. The present absence of agreement arises from the marked variations in diagnostic, conservative, and surgical techniques, different patient populations, the case mix, and reported varying outcomes [41,42,43,44]. This is combined with a substantial heterogeneity of the studies that differ largely regarding the methods and design of the analysis, patients’ features, the preoperative joint condition, measurements, and duration of the follow-up [45,46,47].

While several systematic reviews have been conducted on the topic, significant gaps in the literature remain [10,21,48]. Previous studies have demonstrated variability in surgical techniques, reported outcomes, and inconsistencies in patient selection criteria. Additionally, long-term follow-up data and standardised international guidelines are lacking to support clinical decision making. Addressing these gaps is essential to provide clinicians with robust and evidence-based recommendations for optimal patient management. This systematic review aims to bridge these gaps by providing an updated and comprehensive analysis of the arthroscopic management of FAI, incorporating the most recent data to identify best practises, evaluate patient outcomes, and discuss potential pitfalls. The present systematic review aims to update current evidence on patients who have undergone arthroscopic surgery for FAI, evaluating clinical outcomes, such as pain relief and functional improvement and patient-reported outcomes, including satisfaction and quality of life. This study will also explore advancements in surgical techniques for the management of labral injuries and improvements in eligibility criteria, offering a clearer perspective on the current controversies and future directions in the treatment of FAI, ensuring a thorough examination of the topic and providing valuable insights for clinical practice.

## 2. Materials and Methods

### 2.1. Eligibility Criteria

All the clinical studies investigating the arthroscopic management of FAI were accessed. Only studies published in peer-reviewed journals were considered. According to the authors’ language capabilities, English, Italian, German, Spanish, and French articles were deemed eligible for the present systematic review. In accordance with the 2020 Oxford Centre of Evidence-Based Medicine [49], studies with levels I to III of evidence were considered eligible. Randomised control studies (RCTs), cohort studies, case–control studies, cross-sectional studies and case series were included. Reviews, editorials, opinions, letters, animals, in vitro, computational, biomechanics, and cadaveric studies were not included. Articles which investigated open surgery were not eligible. Only studies with a minimum of six months of follow-up were considered.

### 2.2. Search Strategy

The present systematic review was conducted according to the Preferred Reporting Items for Systematic Reviews and Meta-Analyses: the 2020 PRISMA statement [50]. The following algorithm was used for the literature search:

- Problem: Femoroacetabular impingement.
- Intervention: Surgical management.
- Design: Clinical trial.
- Follow-up: Minimum of 6 months.

In December 2024, the following databases were accessed: PubMed, Web of Science, and Embase, with no additional filters or time constraints. The Medical Subject Headings (MeSH) used for the database search can be found in Appendix A.

### 2.3. Selection and Data Collection

Two authors (F.M. and T.B.) performed the database search. All the resulting titles were screened by hand, and the abstract was accessed if suitable. If a match was found, the full text was examined. If the full text was not accessible or available, the article was not considered for inclusion. The bibliography of the full-text articles was also cross-referenced for inclusion. In case of disagreements, a third senior author (N.M.) made the final decision.

### 2.4. Data Items

Two authors (F.M. and T.B.) performed data extraction. At baseline, the following data were extracted: author, the year of publication and journal, the length of follow-up, the number of patients with a related mean age, and BMI. The data were extracted in Microsoft Office Excel version 16.0 (Microsoft Corporation, Redmond, WA, USA).

### 2.5. Assessment of the Risk of Bias

The risk of bias was evaluated following the guidelines in the Cochrane Handbook for Systematic Reviews of Interventions [51]. Two authors (F.M. and T.B.) independently assessed the bias risk in the extracted studies. Nonrandomised controlled trials (non-RCTs) were evaluated using the risk of bias in Nonrandomised Studies of Interventions (ROBINS-I) tool [52]. Seven domains of potential bias in non-RCTs were assessed. Two domains assessed the possible confounding variables and the nature of patient selection before the start of the comparative intervention. Bias in the classification during the intervention was assessed by a further domain. The final four domains were used to assess the methodological quality after the intervention comparison has been implemented and relate to deviations from previously intended interventions, missing data, the erroneous measurement of outcomes, and bias in the selection of reported outcomes. The figure of the ROBINS-I was elaborated using the Robvis Software (Risk-of-bias VISualization, Riskofbias.info, Bristol, UK) [53].

Randomised controlled trials (RCTs) were checked against the revised risk of bias assessment tool (RoB2) [54,55] of the Cochrane tool for assessing the risk of bias in randomised trials (RoB) [56]. The following biases were considered: from the randomisation process, from deviations from intended interventions, from missing outcome data, in measuring the outcome, and in selecting the reported result.

### 2.6. Synthesis Method

The main author (F.M.) performed the statistical analyses following the recommendations of the Cochrane Handbook for Systematic Reviews of Interventions [51]. For descriptive statistics, the IBM SPSS software version 25 was used. The arithmetic mean and standard deviation were used for continuous data, and the frequency (events/observations) for dichotomic variables.

## 3. Results

### 3.1. Study Selection

**Figure 1:**
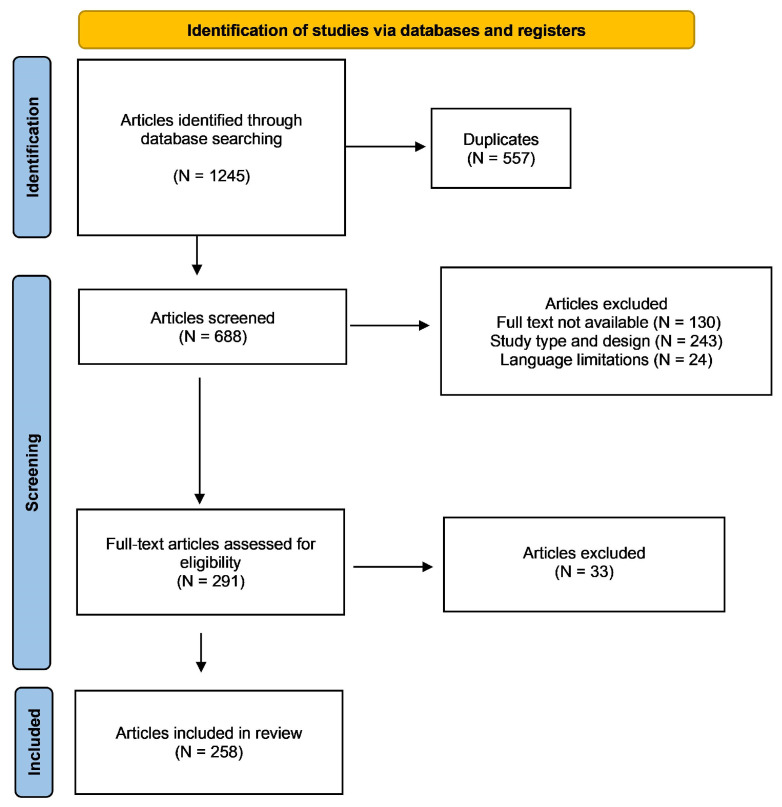
PRISMA flow chart of the literature search.

This systematic review employed a comprehensive search strategy, yielding 1245 articles relevant to the area of investigation. Following deduplication, we screened the abstracts of 688 articles to assess their eligibility. A rigorous exclusion process eliminated 397 articles that did not meet the predefined criteria, primarily due to methodological inconsistencies (N = 243). Language limitations (N = 24) and the inaccessibility of full text (N = 130) further contributed to article exclusion. A full-text review of the remaining 291 articles resulted in the exclusion of an additional 33. Ultimately, this systematic review included a final selection of 258 studies. The results of the literature search are shown in Figure 1.

### 3.2. Risk of Bias Assessment

**Figure 2:**
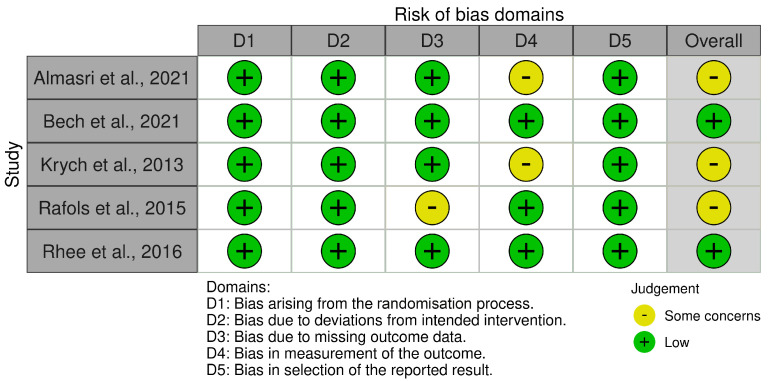
The RoB2 of RCTs [57,58,59,60,61].

Five of the studies included in this systematic review were RCTs. The Cochrane risk of bias assessment tool (ROB 2) was used to evaluate these RCTs. The analysis revealed good comparability between the intervention and control groups at baseline in all studies, suggesting a low risk of bias introduced by the randomisation process. Furthermore, the assessment did not identify noteworthy concerns regarding deviations from the intended intervention protocol or selective outcome reporting. However, two of the five RCTs exhibited a moderate risk of bias in the outcome measurement due to unblinded assessors. Additionally, another trial presented a moderate risk of bias due to missing data. Consequently, three of the five RCTs were judged to have a moderate overall risk of bias, while the remaining two demonstrated a low risk of bias (Figure 2).

**Figure 3:**
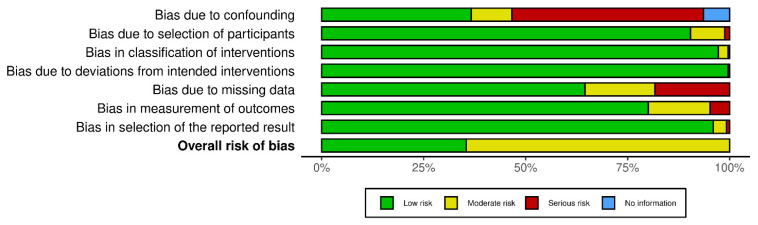
The ROBINS-I of non-RCTs.

The ROBINS-I tool assessed the risk of bias within the non-randomised controlled trials (RCTs). A critical finding emerged in the first domain, where a serious or moderate risk of bias due to confounding was identified in a significant portion of the studies. This highlights a key methodological concern. Conversely, the risk of bias arising from participant selection was generally low across the studies. A low risk of bias was also maintained in nearly all studies for both the classification of interventions and adherence to the intended intervention protocol. However, the domains evaluating post-intervention bias revealed some concerns, particularly regarding missing data. The selection of reported results presented minimal concerns in almost all studies. In conclusion, the ROBINS-I assessment indicated a moderate or low overall risk of bias across the non-RCT studies, suggesting an acceptable methodological quality (Figure 3).

### 3.3. Study Characteristics and Results of Individual Studies

Data from 57,803 patients were retrieved. Of them, 54.3% (31,382 of 57,803 patients) were women. The mean length of follow-up was 34.2 ± 22.7 months. The mean age was 34.7 ± 5.3, and the mean BMI was 25.1 ± 2.0 kg/m^2^. The generalities of the included studies are shown in Appendix B.

## 4. Discussion

FAI is increasingly recognised as a major cause of hip pain, especially among young and active individuals [62]. While short-term benefits of hip arthroscopy, such as pain relief and improved function within the first year post-surgery, are well documented, understanding the factors contributing to sustained long-term success remains crucial. This investigation reviewed current concepts of hip arthroscopy for FAI, demonstrating efficacy and safety across diverse groups of patients. Analysing short-term and long-term outcomes using clinical metrics such as the mHHS and Visual Analogue Scale for Satisfaction offers valuable insights into patient recovery. This article also provides critical guidance on surgical techniques, such as labral repair and capsular management, to optimise patient outcomes. By systematically addressing preoperative, intraoperative, and postoperative factors, this research is thorough and beneficial for clinicians and researchers, contributing significantly to orthopaedic surgery.

### 4.1. Short-Term Outcomes

The short-term outcomes of hip arthroscopy for FAI have been widely studied, demonstrating significant improvements in pain relief and hip function within the first year post-surgery [63,64]. Akpinar et al. [65] analysed the impact of postoperative outcomes on long-term results. In that study, 89 patients were divided into two groups based on their modified Harris Hip Score (mHHS) one year after surgery [65]. A high mHHS at the one-year follow-up was statistically significantly associated with better PROMs and a higher survival rate at the five-year follow-up [65]. This suggests that early improvements in postoperative outcomes can indicate long-term success. A meta-analysis of three RCTs comparing the conservative and arthroscopic treatment of FAI reported superior outcomes at 12 months for patients undergoing hip arthroscopy compared to non-operative treatments [66]. Byrd and Jones [67] and Hufeland et al. [68] documented significant PROM improvements shortly after surgery. Byrd and Jones [67] specifically examined arthroscopic acetabular labral repair in patients over 60, finding that a significant portion could return to their previous activity levels. Beck et al. [69] emphasised the importance of defining meaningful functional improvements. They used the Visual Analogue Scale for Satisfaction, revealing that substantial patient satisfaction could be achieved within two years post-surgery [69]. Basques et al. [70] found that preoperative symptom duration is significantly associated with postoperative outcomes. In their study on 624 patients, those with symptoms lasting less than two years had statistically significantly better outcome scores than those lasting two or more years after two years of follow-up [70]. Patients with shorter symptoms before surgery experienced better outcomes, underscoring the importance of timely intervention [70]. This finding is supported by Kunze et al. [71], who showed that early hip arthroscopy provides superior outcomes compared to delaying surgical treatment beyond six months [71].

### 4.2. Long-Term Outcomes

Long-term outcomes of hip arthroscopy for FAI are more varied and significantly influenced by preoperative joint conditions and specific surgical techniques [72]. Márquez et al. [73] found a statistically significant difference in cumulative survivorship rates among patients with different grades of hip osteoarthritis over 10 years of follow-up. The cumulative survivorship rate at 10 years was 77.8%, with 85.2% for patients with a Tönnis grade of 1 or less compared to 45.4% for patients with a Tönnis grade greater than 1 [73]. This underscores the importance of early intervention and the potential limitations of arthroscopy in patients with more advanced hip degeneration. Domb et al. [74] compared outcomes in patients with Tönnis grade 0 and 1, finding no statistically significant differences in postoperative scores and survivorship between the two groups. Even with early signs of osteoarthritis, patients could achieve positive long-term outcomes similar to those without osteoarthritis [74]. Drager et al. [75] found that patients with a hypotrophic labrum achieved similar long-term outcomes after primary labral repair compared to those with a normal-sized labrum. Labral size might not significantly predict long-term success, provided that labral repair is adequately performed [75].

### 4.3. Sex, Age and Athletic Status

Beck et al. [76] found sex-specific differences in achieving meaningful clinical outcomes after surgery. Females, particularly those with joint hypermobility, may experience less favourable outcomes than males [76]. This can be attributed to differences in hip anatomy, hormonal influences, and the prevalence of conditions such as generalised joint laxity, which is more common in females and can affect the stability of the hip joint post-surgery [76]. However, another recent systematic review showed insufficient high-level evidence supporting sex-specific differences in outcomes after hip arthroscopic surgery [77].

Lin et al. [78] analysed 109 patients who underwent hip arthroplasty and divided them into three groups according to age. The youngest group showed a statistically significant superiority in survival rate compared to the others. However, all the groups showed an improvement in PROMs [78]. Gao et al. [79] demonstrated that even patients aged 50 years or older could achieve positive outcomes with hip arthroscopy, suggesting that age alone should not be a disqualifying factor.

Lindman et al. [80] conducted a 5-year follow-up study on 64 elite athletes. They observed a statistically significant improvement in PROMs, and 54% of the athletes returned to competitive sports [80]. Weber et al. [81] examined 49 collegiate players, and the overall rate of return to sport was 89.7%. A lower rate of return to sport was observed in endurance athletes [81]. A systematic review demonstrated that the return-to-sport rate ranged from 72.7% to 100%, with 74.2–100% of these athletes returning to their preinjury or greater level [82].

### 4.4. Surgical Techniques

The surgical techniques for the arthroscopic treatment of FAI have significantly evolved, aiming to optimise patient outcomes and minimise complications [83]. The key procedures typically involve labral repair or debridement, cam or pincer lesion resection, and capsular management [84,85].

Labral repair and preservation are critical components in most arthroscopic treatments for FAI [86]. Techniques for labral repair vary from simple debridement to more complex repairs using anchors and sutures [57]. Schilders et al. [87], in a long-term follow-up study on labral tears treated arthroscopically, comparing refixation and resection, indicated that refixation provided better long-term outcomes in terms of pain relief and hip function, supporting the importance of preserving and repairing the labrum during surgery [87]. Domb et al. [88] compared labral reconstruction and segmental resection, reporting that labral reconstruction was superior to segmental resection for irreparable labral tears, which led to improved patient outcomes. Chahla et al. [89] emphasised that the length of the acetabular labral tear significantly affects outcomes, with longer tears necessitating meticulous repair to ensure optimal results. Further studies have shown that primary labral reconstruction in patients with FAI, irreparable labral tears, and severe acetabular chondral defects can decrease the risk of conversion to total hip arthroplasty [84]. This underscores the importance of a thorough labral assessment and the application of appropriate surgical techniques to optimise patient outcomes.

Capsular management, including capsulotomy and capsular closure, is crucial in maintaining hip stability and function post-surgery [90]. Complete capsular closure is associated with better outcomes compared to partial closure [91]. Patients undergoing complete capsular closure had higher rates of clinically significant outcome improvement and higher survivorship at a minimum five-year follow-up [92]. Frank et al. [93] also found improved outcomes in patients undergoing T-capsulotomy with complete repair versus partial repair. Another RCT found that capsular closure significantly affects clinical outcomes, with those undergoing closure showing better functional scores [94]. Other capsular management approaches include using a puncture capsulotomy technique with favourable midterm functional outcomes [95]. This technique minimises capsular damage while providing adequate access for the necessary repairs [95].

Osteoplasty, involving the resection of cam and pincer lesions, is a common procedure performed in conjunction with labral repair [96]. Techniques for osteoplasty range from mini-open to fully arthroscopic approaches [97]. Ahmad et al. [98] demonstrated that surgical hip dislocation was more effective than arthroscopy in achieving high degrees of acetabular correction in pincer-type impingement. Büchler et al. [99] compared arthroscopic versus open cam resection and found that both techniques were effective, but arthroscopy was associated with fewer complications and a quicker recovery time. Postoperative osseous correction showed improved results with increasing surgical experience, highlighting the significant learning curve of hip arthroscopy [99]. Similarly, Bedi et al. [100] found that arthroscopic osteoplasty can effectively restore head–neck offset and achieve a similar resection depth and arc to open surgical dislocation for anterior and anterosuperior cam and focal rim impingement deformities. However, the open technique may improve posterosuperior femoral offset loss [100]. Bellotti et al. [101] highlighted the efficacy of a mini-open approach for femoroacetabular impingement, reporting good long-term outcomes and evolved indications for this technique. Advanced techniques in osteoplasty include the use of patient-specific templates and guided osteoplasty [102]. Mihalič et al. [102] reported improved precision and outcomes with these techniques, emphasising their role in optimising surgical accuracy and patient satisfaction. Combined labral repair and femoral osteoplasty in 108 patients improved their PROMs and returned to daily activities without limitations two months post-surgery [26].

### 4.5. Complication

Despite advancements, complications in hip arthroscopy still occur [103]. Common issues include heterotopic ossification, persistent pain, and the need for revision surgery [104]. Jimenez et al. [105] noted higher complication rates in smokers, while Beck et al. [106] found that patients with lumbosacral spine pathology had poorer outcomes. Infections, though rare, require prophylactic antibiotics and aseptic techniques, managed with antibiotics and surgical debridement if necessary [107]. Traction-related complications include nerve injuries, resulting in transient neuropraxia or severe damage [108]. Proper techniques and limiting traction time are crucial [109]. Vascular injuries, although rare, require immediate intervention and careful surgical techniques [110]. Appropriate patient selection and surgical technique are essential to minimise risks and ensure sustained improvements [83].

### 4.6. Clinical Recommendations

Hip arthroscopy for FAI is most effective when performed early, particularly in patients with a Tönnis grade of 1 or less. Delayed intervention is associated with poorer outcomes and an increased risk of osteoarthritis progression. It is essential to consider this surgical approach in younger individuals and older patients with mild osteoarthritic changes, where significant benefits can still be achieved. Labral repair and capsular closure are fundamental components of successful surgical treatment. Labral preservation improves long-term joint function, while complete capsular closure enhances hip stability and recovery. A comprehensive patient evaluation, taking into account age and the degree of joint degeneration, is crucial for optimising outcomes. When performed appropriately, hip arthroscopy provides significant pain relief and functional improvements, ensuring high patient satisfaction and enhancing the quality of life [111].

### 4.7. Limitations

Despite the comprehensive nature of this systematic review, several limitations should be considered. The included studies display substantial heterogeneity in design (prospective vs. retrospective), patient demographics, surgical techniques, and follow-up durations, ranging from 6 months to over 10 years. This variability complicates comparisons and limits generalisability. The absence of standardised surgical protocols, particularly in labral repair, capsular management, and osteoplasty, contributes to inconsistent outcomes across studies. Additionally, variations in surgeon experience may influence reported success rates and complication profiles. Rehabilitation protocols differ widely, from accelerated programmes to conservative approaches, which can impact recovery and patient-reported outcomes. A bias assessment using the ROBINS-I tool indicated a low-to-moderate risk across most studies, with confounding factors and missing data identified as primary concerns. Language restrictions may have introduced selection bias by excluding studies published in other languages. The predominance of retrospective studies over randomised controlled trials further limits the strength of the evidence. Future research should focus on developing standardised surgical and rehabilitation protocols and personalising surgical strategies based on individual patient characteristics such as age, activity level, and preoperative joint conditions to improve long-term outcomes.

## Data Availability

Data are contained within this article.

## Appendix A. Searching Strategy

“Acetabulum”[Mesh] OR “Acetabulum/injuries”[Mesh] OR “Acetabulum/pathology”[Mesh] OR “Arthralgia/etiology”[Mesh] OR “Cartilage/physiopathology”[Mesh] OR “Femoracetabular Impingement”[Mesh] OR “Femoracetabular Impingement/diagnosis”[Mesh] OR “Femoracetabular Impingement/etiology”[Mesh] OR “Femoracetabular Impingement/pathology”[Mesh] OR “Femoracetabular Impingement/physiopathology”[Mesh] OR “Hip”[Mesh] OR “Hip Joint/pathology”[Mesh] OR “Joint Diseases/pathology”[Mesh] OR “Pain”[Mesh] OR Acetabulum OR cam OR Cam impingement OR cam lesion OR cam type OR FAI OR FAI syndrome OR FAIS OR Femoracetabular impingement OR Femoro-acetabular impingement OR Hip joint OR hip pain OR Pincer OR pincer hip OR Pincer impingement OR pincer type **AND** “Acetabulum/surgery”[Mesh] OR “Arthralgia/surgery”[Mesh] OR “Arthroscopy”[Mesh] OR “Arthroscopy/methods”[Mesh] OR “Arthroscopy/standards”[Mesh] OR “Cartilage/surgery”[Mesh] OR “Debridement”[Mesh] OR “Femoracetabular Impingement/surgery”[Mesh] OR “Hip Joint/surgery”[Mesh] OR “Joint Diseases/surgery”[Mesh] OR acetabular labral refixation OR acetabular labral segmental reconstruction OR arthroscopic labral reconstruction OR arthroscopic surgery OR arthroscopic surgical procedures OR Arthroscopy OR bone marrow concentrate OR labral reconstruction OR labral repair OR Reconstruction OR surgery **AND** “Pain Measurement/methods”[Mesh] OR “Patient Outcome Assessment”[Mesh] OR “Patient Reported Outcome Measures”[Mesh] OR “Patient Satisfaction”[Mesh] OR “Visual Analog Scale”[Mesh] OR patient outcomes OR Patient Reported Outcome Measures OR Treatment outcome OR VAS OR visual analog scale

**SUMMARY:** (“Acetabulum”[Mesh] OR “Acetabulum/injuries”[Mesh] OR “Acetabulum/pathology”[Mesh] OR “Arthralgia/etiology”[Mesh] OR “Cartilage/physiopathology”[Mesh] OR “Femoracetabular Impingement”[Mesh] OR “Femoracetabular Impingement/diagnosis”[Mesh] OR “Femoracetabular Impingement/etiology”[Mesh] OR “Femoracetabular Impingement/pathology”[Mesh] OR “Femoracetabular Impingement/physiopathology”[Mesh] OR “Hip”[Mesh] OR “Hip Joint/pathology”[Mesh] OR “Joint Diseases/pathology”[Mesh] OR “Pain”[Mesh] OR Acetabulum OR cam OR Cam impingement OR cam lesion OR cam type OR FAI OR FAI syndrome OR FAIS OR Femoracetabular impingement OR Femoro-acetabular impingement OR Hip joint OR hip pain OR Pincer OR pincer hip OR Pincer impingement OR pincer type) AND (“Acetabulum/surgery”[Mesh] OR “Arthralgia/surgery”[Mesh] OR “Arthroscopy”[Mesh] OR “Arthroscopy/methods”[Mesh] OR “Arthroscopy/standards”[Mesh] OR “Cartilage/surgery”[Mesh] OR “Debridement”[Mesh] OR “Femoracetabular Impingement/surgery”[Mesh] OR “Hip Joint/surgery”[Mesh] OR “Joint Diseases/surgery”[Mesh] OR acetabular labral refixation OR acetabular labral segmental reconstruction OR arthroscopic labral reconstruction OR arthroscopic surgery OR arthroscopic surgical procedures OR Arthroscopy OR bone marrow concentrate OR labral reconstruction OR labral repair OR Reconstruction OR surgery) AND (“Pain Measurement/methods”[Mesh] OR “Patient Outcome Assessment”[Mesh] OR “Patient Reported Outcome Measures”[Mesh] OR “Patient Satisfaction”[Mesh] OR “Visual Analog Scale”[Mesh] OR patient outcomes OR Patient Reported Outcome Measures OR Treatment outcome OR VAS OR visual analog scale)

## Appendix B

**Table A1:** Generalities and patient demographics of the included studies.

Author and Year	Journal	Design	Follow-Up (Months)	Patients	Women	Mean Age
Abrahamson et al., 2020 [112]	*J*. *Exp*. *Orthop*.	Prospective	23.4	135	127	30.0
				416		
Aguilera-Bohorquez, 2019 [113]	*Arthroscopy*	Retrospective	12	17	9	34.6
Aguilera-Bohorquez, 2020 [114]	*Rev*. *Bras*. *Ortop*.	Retrospective	12	34	23	65.5
				34	23	30.6
Ahmad et al., 2019 [98]	*Orthop*. *Traumatol*. *Surg*. *Res*.	Retrospective	24	82	14	37.9
					27	33.5
Akpinar et al., 2020 [65]	*Am*. *J*. *Sports Med*.	Retrospective	73.7	45	34	45.3
				44	26	41.2
Almasri et al., 2021 [58]	*Knee Surg*. *Sports Traumatol*. *Arthrosc*.	Prospective	12	108	42	36.7
Alvarez, Mardones, 2010 [115]	*Cartilage*	Retrospective		76	58	29.7
Atzmon et al., 2019 [116]	*J*. *Hip*. *Preserv*. *Surg*.	Retrospective	60.7	29	13	37.6
			40.4	35	14	38.1
Ayeni et al., 2021 [117]	*Am*. *J*. *Sports Med*.		24	108	42	36.7
				106	39	35.4
Bali et al., 2014 [118]	*Bone Joint J*.	Retrospective	41	8	2	17.8
Bardakos et al., 2008 [119]	*J*. *Bone Joint Surg*. *Br*.	Retrospective	12	24	10	33.0
				47	24	35.0
Basques et al., 2019 [70]	*Am*. *J*. *Sports Med*.	Retrospective	24	389	243	33.0
				235	164	35.5
Bech et al., 2021 [59]	*Hip*. *Int*.	Prospective	12	58	35	35.5
				58	39	33.5
Beck et al., 2019 [120]	*Am*. *J*. *Sports Med*.	Retrospective	24	112	81	33.6
				224	153	33.7
Beck et al., 2020 [106]	*Am*. *J*. *Sports Med*.	Retrospective	24	83	48	39.9
				166	114	40.0
Beck et al., 2019 [69]	*Arthroscopy*	Retrospective	24	335	232	32.8
Beck et al., 2021 [92]	*Arthroscopy*	Retrospective	71.9	25	18	35.0
			66.7	75	49	35.1
Beck et al., 2021 [76]	*Arthroscopy*	Retrospective	60	25		37.4
				25		
				25		
				25	25	
				25	25	
				25	25	
Bedi et al., 2011 [121]	*Am*. *J*. *Sports Med*.	Prospective	10.9	10		25.9
Bedi et al., 2011 [100]	*Am*. *J*. *Sports Med*.	Retrospective		30		30.0
				30		
Bellotti et al., 2016 [101]	*Hip*. *Int*.	Retrospective	104.4	296		
Bloom et al., 2020 [122]	*Am*. *J*. *Sports Med*.	Retrospective	24	44	44	25.2
				74	74	37.1
				76	76	55.2
Bodendorfer et al., 2021 [123]	*Orthop*. *J*. *Sports Med*.	Retrospective	24.5	372	231	37.7
				124	77	38.9
Bolia et al., 2019 [124]	*Arthroscopy*	Retrospective	87.6	42	18	38.0
			76.8	84	32	
Botser et al., 2014 [125]	*Am*. *J*. *Orthop*.	Prospective	14.3	18	23	20.1
			16.2	5		18.1
Brick et al., 2020 [126]	*Am*. *J*. *Sports Med*.	Prospective	24	93	57	34.5
				93	57	34.5
Briggs et al., 2018 [127]	*Knee Surg*. *Sports Traumatol*. *Arthrosc*.	Retrospective	61.5	159		40.0
				71		
Bryan et al., 2016 [63]	*Am*. *J*. *Sports Med*.	Prospective	51.6	174	138	37.0
			46.8	27		61.0
Büchler et al., 2013 [99]	*Arthroscopy*	Retrospective	16.7	66	49	33.8
				135	44	31.2
Büchler et al., 2021 [128]	*Clin*. *Orthop*. *Relat*. *Res*.	Retrospective	132	50	45	33.0
Byrd et al., 2016 [129]	*Arthroscopy*	Retrospective	30	108	65	15.9
				122	51	36.8
Byrd et al., 2019 [67]	*Arthroscopy*		18.9	21	10	63.2
				21	10	35.8
Byrd, Jones, 2009 [130]	*Arthroscopy*	Prospective	120	26	13	46.0
Byrd, Jones, 2009 [131]	*Clin*. *Orthop*. *Relat*. *Res*.	Prospective	16	200	62	33.0
				200	62	33.4
Byrd, Jones, 2011 [132]	*Am*. *J*. *Sports Med*.	Prospective	19	200	52	28.6
Cancienne et al., 2019 [133]	*Am*. *J*. *Sports Med*.	Retrospective	24	40	24	35.5
				80	49	36.1
Capogna et al., 2016 [134]	*Arthroscopy*	Prospective	26.4	42	34	65.8
Chahla et al., 2019 [89]	*Am*. *J*. *Sports Med*.	Prospective	29.5	600	218	32.4
					169	34.6
Chahla et al., 2019 [135]	*Am*. *J*. *Sports Med*.	Prospective	27.8	493	346	34.0
				92	45	35.4
				49	26	37.4
Charles et al., 2021 [136]	*PLoS ONE*	Retrospective	53	54	25	35.0
Cho, 2015 [137]	*Hip*. *Pelvis*.	Retrospective	24	11	7	45.0
Clohisy et al., 2010 [138]	*Clin*. *Orthop*. *Relat*. *Res*.	Prospective	26.4	35		34.0
Clohisy et al., 2013 [139]	*Am*. *J*. *Sports Med*.	Prospective		1076	592	28.4
Cohen et al., 2012 [140]	*Am*. *J*. *Sports Med*.	Retrospective	22	59	21	32.0
Comba et al., 2016 [141]	*Muscles Ligaments Tendons J*.	Prospective	91	42	15	38.0
Cong et al., 2022 [142]	*J*. *Clin*. *Med*.	Retrospective	10.4	22	14	38.3
De Lucas Villarrubia, 2021 [143]	*Arthroscopy*	Retrospective	29	25	6	40.5
Degen et al., 2017 [144]	*Arthroscopy*	Retrospective	36.1	34	16	16.0
			34.1	296	137	31.0
Domb et al., 2014 [88]	*Am*. *J*. *Sports Med*.	Retrospective	26.4	11	4	33.0
			30	22	8	38.8
Domb et al., 2015 [145]	*Arthroscopy*	Prospective	32.8	52	34	54.8
			33.1	52	34	20.3
Domb et al., 2017 [74]	*Am*. *J*. *Sports Med*.	Retrospective	70	62	37	41.9
			72.6	62	37	42.3
Domb et al., 2020 [146]	*Am*. *J*. *Sports Med*.	Prospective	50.77	98	62	40.4
				98	62	40.5
				98	62	40.7
Drager et al., 2020 [75]	*Arthroscopy*	Retrospective	12	173	115	31.3
				173	134	31.5
Eberlin et al., 2023 [95]	*Orthop*. *J*. *Sports Med*.	Prospective	30.4	163	84	37.9
Ellis et al., 2021 [147]	*Arch*. *Orthop*. *Trauma Surg*.	Prospective	6	22	22	20.0
				22	22	22.6
Ernat et al., 2019 [148]	*Arthroscopy*	Retrospective	42	182	48	30.4
Essilfie et al., 2020 [149]	*Arthroscopy*	Prospective	24	84	28	36.0
				84	56	36.2
Ezechieli et al., 2016 [150]	*Technol. Health Care*	Prospective	15	72	34	32.1
						28.5
Fabricant et al., 2015 [151]	*J*. *Bone Joint Surg*. *Am*.	Retrospective	21	37	15	28.0
				149	75	30.0
				57	33	29.0
Feghhi et al., 2020 [152]	*Am*. *J*. *Sports Med*.	Retrospective	44.4	18	14	28.0
			26.1	19	14	27.3
Ferrer-Rivero et al., 2024 [153]	*J*. *ISAKOS*	Retrospective	32.4	11	11	32.0
				22	22	
Filan, Carton, 2020 [154]	*Arthroscopy*	Retrospective	27.6	508	80	28.5
			30	458	57	27.6
Filan, Carton, 2021 [155]	*Arthroscopy*	Retrospective	28.8	486		25.9
Flores et al., 2018 [156]	*Orthop*. *J*. *Sports Med*.	Prospective	15.5	30	15	37.2
			13.1	30	13	35.3
Flores et al., 2020 [157]	*Orthop*. *J*. *Sports Med*.	Prospective	24	72	72	34.2
				59		35.8
Foo et al., 2021 [158]	*J*. *Hip*. *Preserv*. *Surg*.	Prospective	24	58	26	36.1
				48	21	35.9
Forster-Horvath et al., 2021 [159]	*Arthroscopy*	Retrospective	55	78		33.1
Frank et al., 2014 [93]	*Am*. *J*. *Sports Med*.	Retrospective	29.9	32	20	32.9
				32	20	32.7
Frank et al., 2019 [160]	*Orthop*. *J*. *Sports Med*.	Retrospective	31.2	97	97	36.0
				97	97	37.8
Fukui et al., 2015 [161]	*Arthroscopy*	Retrospective	40	100	51	35.0
Fukui et al., 2015 [162]	*Bone Joint J*.	Retrospective	42	28	12	34.0
Gao et al., 2019 [163]	*Chin*. *Med*. *J*.	Retrospective	22.6	13	7	37.5
			23.1	229	95	36.2
Gao et al., 2020 [79]	*J*. *Orthop*. *Surg*.	Prospective	24	27	12	57.0
Gao et al., 2022 [164]	*BMC Musculoskelet*. *Disord*.	Retrospective	28.1	37	38	36.3
				34		
Gao et al., 2022 [165]	*J*. *Orthop*. *Surg*. *Res*.		14.3	194	106	37.1
		
Gebhardt et al., 2023 [166]	*Cartilage*	Prospective	24	11		29.4
Gedouin et al., 2010 [167]	*Orthop*. *Traumatol*. *Surg*. *Res*.	Retrospective	15.6	38		36.0
Gedouin et al., 2010 [168]	*Orthop*. *Traumatol*. *Surg*. *Res*.	Prospective	10	110	32	31.0
Gicquel et al., 2014 [8]	*Orthop*. *Traumatol*. *Surg*. *Res*.	Prospective	55.2	51	32	
Giordano et al., 2020 [169]	*Am*. *J*. *Sports Med*.	Retrospective	75.2	336	210	37.7
				42		
				9		
				51		
Glaws et al., 2019 [170]	*J*. *Sport Rehabil*.	Cohort study?	6	16	7	23.4
				12	8	27.4
Goyal T., 2018 [171]	*SICOT-J*.	Retrospective	24	15		27.5
Gupta et al., 2014 [172]	*Am*. *J*. *Sports Med*.	Prospective	28.32	47	19	37.2
Gupta et al., 2015 [173]	*Am*. *J*. *Sports Med*.	Prospective	28.98	595	367	38.0
Gürsan et al., 2023 [174]	*J*. *Hip*. *Preserv*. *Surg*.	Retrospective	80.4	52	19	33.9
Ha et al., 2020 [175]	*J*. *Orthop*. *Surg*.	Retrospective	24	62	6	31.4
Haefeli et al., 2017 [176]	*Clin*. *Orthop*. *Relat*. *Res*.	Retrospective	79	50	46	35.0
Hartigan et al., 2017 [177]	*J*. *Hip*. *Preserv*. *Surg*.	Retrospective	42	69	24	43.6
Hartmann, Gunther, 2009 [178]	*Arch*. *Orthop*. *Trauma Surg*.	Retrospective	15	33	16	31.0
Hartwell et al., 2021 [179]	*Arthroscopy*	Retrospective	89.1	86	61	39.8
Haskel et al., 2020 [180]	*Am*. *J*. *Sports Med*.	Retrospective	24	38	29	48.2
				111	84	48.1
Hassebrock et al., 2019 [181]	*Arthroscopy*	Retrospective	63.54	133	86	32.0
			28.33	133	86	33.0
Hassebrock et al., 2020 [182]	*Am*. *J*. *Sports Med*.	Retrospective	26	22	6	22.0
				28	12	19.0
Hassebrock et al., 2020 [183]	*Am*. *J*. *Sports Med*.	Retrospective	24	49	17	19.4
				62	18	18.6
Hatakeyama et al., 2017 [184]	*Am*. *J*. *Sports Med*.	Retrospective	42.5	34	20	20.0
				11	10	47.0
Haviv et al., 2010 [185]	*J*. *Bone Joint Surg*. *Br.*	Retrospective	22	166	34	32.2
						35.2
						43.3
Haviv, O’Donnell, 2010 [186]	*Orthopedics*	Prospective	26	82		29.0
Haws et al., 2022 [187]	*J*. *Hip*. *Preserv*. *Surg*.	Retrospective	12	22	14	21.6
				74	51	23.3
Hevesi et al., 2018 [188]	*Am*. *J*. *Sports Med*.	Prospective	68.4	48	27	31.8
				96	49	31.4
Holleyman et al., 2023 [189]	*Knee Surg*. *Sports Traumatol*. *Arthrosc*.	Retrospective	12	2971	1575	35.8
				462	343	35.2
				1530	782	35.3
Honda et al., 2019 [190]	*Knee Surg*. *Sports Traumatol*. *Arthrosc*.	Retrospective	32.2	57	26	30.9
				18	12	56.7
				9	3	73.5
Horisberger et al., 2010 [191]	*Arthroscopy*	Prospective	36	20	4	47.3
Horisberger et al., 2010 [192]	*Clin*. *Orthop*. *Relat*. *Res*.	Prospective	27.6	88	28	40.9
Hufeland et al., 2016 [68]	*Arch*. *Orthop*. *Trauma Surg*.	Retrospective	66.3	44	20	34.3
Ilizaturri et al., 2007	*J*. *Bone Joint Surg*.	Prospective	30	13	7	30.6
Ilizaturri et al., 2008	*J*. *Arthroplast.*	Prospective		19	8	34.0
Impellizzeri et al., 2012 [193]	*Osteoarthr. Cartil.*	Prospective	6	102	57	35.9
Jimenez et al., 2022 [105]	*Orthop*. *J*. *Sports Med*.	Retrospective	39.9	20		41.4
				60		42.5
Ju et al., 2023 [194]	*Orthop*. *Surg*.	Retrospective		15	5	37.1
				26	12	40.2
Kaplan et al., 2021 [195]	*Arthroscopy*	Retrospective	32.4	42	25	39.1
				19	11	36.0
Knapik et al., 2020 [196]	*Am*. *J*. *Sports Med*.	Retrospective	24	63	41	31.7
				45	29	25.2
Kollmorgen et al., 2024 [197]	*Arthroscopy*	Retrospective		25	13	26.5
				25	14	25.5
Krych et al., 2013 [57]	*Arthroscopy*	Prospective	32	18	18	38.0
				18	18	39.0
Kunze et al., 2019 [71]	*Am*. *J*. *Sports Med*.	Retrospective	30.8	250	721	32.3
				265		
				284		
				295		
Kunze et al., 2019 [198]	*J*. *Hip*. *Preserv*. *Surg*.	Retrospective	31.2	204	121	34.7
				102	56	34.9
Kunze et al., 2020 [199]	*Arthroscopy*	Retrospective	60	190	115	32.3
				120	75	36.2
LaFrance et al., 2015 [96]	*J*. *Hip*. *Preserv*. *Surg*.	Prospective	18.5	20		34.4
			23.3	15		34.9
Lall et al., 2019 [200]	*Arthroscopy*	Retrospective	70.6	59	39	37.4
			71.6	59	39	37.2
Larson et al., 2012 [201]	*Am*. *J*. *Sports Med*.	Prospective	42	44	17	32.0
				50	21	28.0
Larson et al., 2019 [202]	*Arthroscopy*	Retrospective	39.8	28	7	15.9
Larson, Giveans, 2008 [203]	*Arthroscopy*	Prospective	9.9	96	42	34.7
Larson, Giveans, 2009 [204]	*Arthroscopy*	Retrospective	21.4	34	9	31.0
			16.5	37	14	27.0
Laude et al., 2009 [205]	*Clin*. *Orthop*. *Relat*. *Res*.	Retrospective	58.3	91	47	33.4
Laurito et al., 2021 [206]	*Acta Ortop*. *Bras*.	Retrospective	17	194	63	39.0
Lee et al., 2019 [207]	*Clin*. *Orthop*. *Surg*.	Retrospective	92.4	41	20	34.6
Lee et al., 2021 [208]	*J*. *Hip*. *Preserv*. *Surg*.	Retrospective	131.5	28	9	36.5
			135	87	27	34.0
Lee et al., 2022 [209]	*Orthop*. *J*. *Sports Med*.	Retrospective	38.6	84	62	45.0
				84	58	
Lee et al., 2023 [210]	*Orthop*. *J*. *Sports Med*.	Retrospective	24	14	3	35.2
				23	15	41.6
				38	11	40.2
Levy et al., 2016 [211]	*Am*. *J*. *Sports Med*.	Retrospective	24	51	29	26.3
Levy et al., 2017 [212]	*Am*. *J*. *Sports Med*.	Retrospective	31.2	28	18	35.8
				56	36	35.2
Lin et al., 2020 [78]	*Am*. *J*. *Sports Med*.	Retrospective	74.2	36	25	27.7
				37	24	41.5
				36	26	60.2
Lin et al., 2021 [213]	*Arthroscopy*	Retrospective	24	152	89	39.2
				21	18	43.5
Lincoln et al., 2009 [214]	*Arthroscopy*	Retrospective	24	14	4	37.0
Lindman et al., 2020 [80]	*Am*. *J*. *Sports Med*.	Prospective	60	64	12	24.0
Lindman et al., 2021 [62]	*Orthop*. *J*. *Sports Med*.	Prospective	24	172	3	28.0
Litrenta et al., 2019 [215]	*J*. *Pediatr*. *Orthop*.	Retrospective	50.4	43	38	16.1
Litrenta et al., 2020 [216]	*J*. *Pediatr*. *Orthop*.	Retrospective	45.2	69		15.9
Maimaitimin et al., 2022 [217]	*Orthop*. *J*. *Sports Med*.	Retrospective	39.4	272	123	37.2
Maldonado et al., 2018 [218]	*Am*. *J*. *Sports Med*.	Retrospective	42.5	307	289	27.8
			43.9	354	277	34.1
Maldonado et al., 2019 [219]	*Arthroscopy*	Retrospective	59.7	18	9	41.2
			51.4	54	27	41.1
Maldonado et al., 2019 [84]	*J*. *Hip*. *Preserv*. *Surg*.	Retrospective	42.4	38	16	43.2
			54.3	38	16	43.7
Maldonado et al., 2020 [220]	*Am*. *J*. *Sports Med*.	Retrospective	72	45	34	39.0
			65.31	45	33	38.3
Maldonado et al., 2020 [221]	*BMC Musculoskelet*. *Disord*.	Retrospective	66.8	25	14	41.4
		
Mardones et al., 2016 [222]	*Muscles Ligaments Tendons*. *J*.	Retrospective	48	15		33.5
Marom et al., 2023 [223]	*Knee Surg*. *Sports Traumatol*. *Arthrosc*.	Retrospective	72	119	51	21.6
Martinez et al., 2019 [224]	*J*. *Orthop*.	Prospective	6	65	25	38.8
				45	18	37.6
Martinez et al., 2020 [225]	*Arthrosc*. *Sports Med*. *Rehabil*.	Retrospective	24	76	22	38.8
				50	2	33.7
				36	22	36.1
				18	3	36.8
Martinez et al., 2023 [73]	*Rev*. *Esp*. *Cir*. *Ortop*. *Traumatol*.	Retrospective	132	17	2	47.8
				54	9	40.6
Martinot et al., 2020 [226]	*Orthop*. *Traumatol*. *Surg*. *Res*.	Retrospective	55.2	55	14	32.3
				36	6	31.4
Matsuda et al., 2015 [85]	*J*. *Hip*. *Preserv*. *Surg*.	Prospective	24	127	67	39.8
				18	6	37.2
Matsuda et al., 2021 [227]	*Arthroscopy*	Retrospective	24.6	16	10	38.0
				76	15	35.0
				1301	898	35.0
May et al., 2020 [228]	*Orthop*. *Traumatol*. *Surg*. *Res*.	Prospective	14.57	42	14	35.4
			15.86	79	58	32.8
				66		
McConkey et al., 2019 [229]	*J*. *Pediatr*. *Orthop*.	Prospective	24	12	5	15.7
				12	5	16.5
Menge et al., 2021 [230]	*Am*. *J*. *Sports Med*.	Prospective	134	60		16.0
					49	16.0
Mercier et al., 2019 [231]	*Orthop*. *Traumatol*. *Surg*. *Res*.	Retrospective	29.6	20	6	32.5
			31.4	23	5	33.5
Migliorini et al., 2023 [26]	*J*. *Orthop*. *Surg*. *Res*.		72.8	108	46	41.5
Mihalic et al., 2021 [102]	*Int*. *Orthop*.	Retrospective	50	5		37.0
Mohan et al., 2017 [232]	*Arthroscopy*	Retrospective	34	50	33	17.8
Moon et al., 2020 [233]	*Arthroscopy*	Retrospective	62.4	73	15	34.4
Moriya et al., 2017 [234]	*J*. *Orthop*. *Surg*. *Res*.	Retrospective	28	23	17	59.3
Mortensen et al., 2023 [235]	*Arthrosc*. *Sports Med*. *Rehabil*.	Prospective		57	32	31.2
Mullins et al., 2019 [236]	*Knee Surg*. *Sports Traumatol*. *Arthrosc*.	Prospective	12	47		24.6
				32		24.3
Mullins et al., 2021 [237]	*Orthop*. *J*. *Sports Med*.	Prospective	27.1	760	50	26.3
Nakashima et al., 2019 [238]	*Am*. *J*. *Sports Med*.	Retrospective	37	25	7	52.6
			31.9	126	73	36.5
Nakashima et al., 2021 [239]	*Clin*. *J*. *Sport Med*.	Retrospective	35.1	79	45	51.3
			29.7	18	9	56.3
Nawabi et al., 2016 [240]	*Am*. *J*. *Sports Med*.	Prospective	31.3	46	22	29.8
				131	73	29.6
Nguyen et al., 2020 [241]	*Am*. *J*. *Sports Med*.	Prospective	24	166	91	35.3
Nho et al., 2011 [242]	*Am*. *J*. *Sports Med*.	Retrospective	27	47	13	22.8
				47	13	22.8
Nho et al., 2019 [83]	*Am*. *J*. *Sports Med*.	Prospective	26.76	901	588	33.3
				34		
Nielsen et al., 2014 [243]	*BMC Musculoskelet*. *Disord*.	Prospective	24	117	69	37.0
Nwachukwu et al., 2020 [244]	*Am*. *J*. *Sports Med*.	Prospective	60	283	179	34.2
Öhlin et al., 2019 [245]	*Knee Surg*. *Sports Traumatol*. *Arthrosc*.	Prospective	60	184	74	38.0
Ohlsen et al., 2024 [246]	*Orthop*. *J*. *Sports Med*.	Retrospective	13.9	22	21	31.1
				22	21	27.8
Olivero et al., 2023 [247]	*SICOT-J*.	Retrospective	12	54	24	35.0
				44	12	39.9
O’Reilly et al., 2022 [97]	*Arthrosc*. *Sports Med*. *Rehabil*.	Retrospective	29.3	104	104	26.9
			29.97	56		23.0
Ortiz-Declet et al., 2019 [248]	*Arthroscopy*	Retrospective	47.4	34	19	20.8
Owens et al., 2022 [249]	*Orthop*. *J*. *Sports Med*.	Prospective	36.1	30	16	29.4
			34.5	60	33	27.5
Owens et al., 2022 [250]	*Orthop*. *J*. *Sports Med*.	Retrospective	30.2	29		40.3
			27.6	29	29	40.5
Özbek et al., 2021 [251]	*Acta Orthop*. *Traumatol*. *Turc*.	Prospective	24	34	20	32.3
Palmer et al., 2012 [252]	*Arthroscopy*	Retrospective	46	185	102	40.2
Pansard et al., 2021 [253]	*Orthop*. *Traumatol*. *Surg*. *Res*.	Prospective	12	42	74	33.3
				10		
				145		
Parvaresh et al., 2020 [254]	*Am*. *J*. *Sports Med*.	Retrospective	62.1	60	33	37.2
				40	22	37.3
				20	11	38.3
				20	11	37.7
Parvaresh et al., 2021 [255]	*Am*. *J*. *Sports Med*.	Retrospective	24	329	218	32.5
				329	218	32.6
Parvaresh et al., 2021 [256]	*Phys*. *Ther*. *Sport*	Retrospective	24	23	14	36.2
Perets et al., 2017 [257]	*Arthroscopy*	Prospective	35.7	10	10	14.7
Perets et al., 2018 [258]	*Arthroscopy*	Retrospective	49.1	60	46	19.5
Perets et al., 2018 [259]	*J*. *Bone Joint Surg*. *Am*.	Retrospective	71.6	74	45	44.2
			71.3	74	45	
Perets et al., 2019 [260]	*Arthroscopy*	Retrospective	69.3	57	50	26.5
			71.7	57	50	28.0
Perets et al., 2019 [261]	*J*. *Am*. *Acad*. *Orthop*. *Surg*.	Retrospective	68.7	407	219	32.4
Philippon et al., 2007 [18]	*Knee Surg*. *Sports Traumatol*. *Arthrosc*.	Retrospective	19.2	45	3	31.0
Philippon et al., 2009 [262]	*J*. *Bone Joint Surg*. *Br*.	Prospective	27.6	112	62	40.6
Philippon et al., 2010 [263]	*Am*. *J*. *Sports Med*.	Retrospective	24	28		27.0
Philippon et al., 2012 [107]	*Arthroscopy*	Retrospective	37.5	153	81	57.0
Philippon et al., 2012 [264]	*Arthroscopy*	Retrospective	36	60	41	15.0
Polesello et al., 2009 [265]	*Rev*. *Bras*. *Ortop*.	Retrospective	27	28	9	34.0
Polesello et al., 2014 [266]	*Hip*. *Int*.	Retrospective	73.2	24	3	34.6
Pontiff et al., 2016 [267]	*Int*. *J*. *Sports Phys*. *Ther*.	Retrospective	6	35	35	25.7
				131	131	33.5
Rafols et al., 2015 [60]	*Arthroscopy*	Prospective	24	30		35.3
				27		
Ramos et al., 2020 [268]	*J*. *Hip*. *Preserv*. *Surg*.	Retrospective	19.2	10		45.0
Rhee et al., 2016 [61]	*Arch*. *Orthop*. *Trauma*. *Surg*.	Prospective	32.3	19	9	33.8
			31.8	18	13	34.6
Riedl et al., 2020 [269]	*J*. *Clin*. *Med*.	Retrospective	33.4	14	7	36.2
			26.3	26	10	36.1
Rivera et al., 2019 [270]	*J*. *Invest*. *Surg*.	Retrospective	24	40	13	41.8
				40	14	42.5
Rosinsky et al., 2019 [271]	*Am*. *J*. *Sports Med*.	Retrospective	35	44	19	17.3
				38	30	
Said et al., 2019 [272]	*SICOT-J*.	Retrospective	32.8	85	13	30.3
		
		
Saito et al., 2019 [273]	*Am*. *J*. *Sports Med*.	Retrospective	24	25		17.5
						22.0
						18.5
						17.0
Saks et al., 2021 [274]	*Arthroscopy*	Retrospective	63.6	41	27	38.9
			65.6	41	25	37.2
Sanders et al., 2016 [275]	*Knee Surg*. *Sports Traumatol*. *Arthrosc*.	Retrospective	30	46	31	42.4
Sansone et al., 2015 [276]	*J*. *Hip*. *Preserv*. *Surg*.	Prospective	12.8	75	18	47.0
Sansone et al., 2015 [277]	*Orthop*. *J*. *Sports Med*.	Retrospective	12.3	85	21	25.0
Sansone et al., 2016 [278]	*Scand*. *J*. *Med*. *Sci*. *Sports*	Prospective	25.4	289	99	37.0
Scanaliato et al., 2020 [279]	*Arthroscopy*	Retrospective	24.3	30	17	30.4
Schilders et al., 2011 [87]	*J*. *Bone Joint Surg*. *Br*.	Retrospective	24	96	25	37.0
		
Seijas et al., 2023 [104]	*Surg*. *J*. (*N*. *Y*.)	Retrospective	24	15	2	42.7
				50	16	39.4
Shao et al., 2022 [280]	*Orthop*. *Surg*.	Retrospective	39.09	33	15	39.3
			38.63	167	82	37.4
Shuang et al., 2024 [281]	*Orthop*. *J*. *Sports Med*.	Retrospective	24	63	56	37.0
Singh et al., 2010 [282]	*Arthroscopy*	Prospective	22	24		22.0
Skendzel et al., 2014 [283]	*Am*. *J*. *Sports Med*.	Prospective	73	383		37.0
				63		46.0
Skowronek et al., 2017 [284]	*Indian*. *J*. *Orthop*.	Retrospective	45	39	14	29.3
Snaebjörnsson et al., 2022 [285]	*Arthroscopy*	Prospective	24	84	17	19.8
Sobti et al., 2020 [286]	*J*. *Hip*. *Preserv*. *Surg*.	Retrospective	12	46	27	45.0
				65	40	45.0
Soriano et al., 2021 [287]	*Arthroscopy*	Retrospective	24	37	19	36.8
				37	19	36.7
Stake et al., 2013 [288]	*Am*. *J*. *Sports Med*.	Prospective	24	20	3	39.0
				20	3	
Stone et al., 2019 [289]	*Am*. *J*. *Sports Med*.	Retrospective	24	514	334	32.4
				174	115	35.9
Stone et al., 2019 [290]	*Arthroscopy*	Retrospective	24	464	437	31.6
				60		
				47		
				10		
Stone et al., 2019 [291]	*J*. *Hip*. *Preserv*. *Surg*.	Prospective	29.3	100	100	22.7
				25	25	18.0
Sugarman et al., 2021 [94]	*Orthop*. *J*. *Sports Med*.	Prospective	24	28	8	33.7
				28	22	45.0
Sutton et al., 2021 [292]	*Clin*. *Orthop*. *Relat*. *Res*.	Retrospective	48	60	43	27.0
				20	12	27.0
Thaunat et al., 2020 [32]	*Orthop*. *Traumatol*. *Surg*. *Res*.	Retrospective	34.71	39	9	28.8
				25	4	28.5
Tjong et al., 2016 [293]	*Orthop*. *J*. *Sports Med*.	Retrospective	24	13	7	44.0
				10	8	43.7
Torabian et al., 2023 [294]	*Am*. *J*. *Sports Med*.	Retrospective	38.2	70	37	39.4
			39.2	87	40	35.8
Torabian et al., 2024 [295]	*Am*. *J*. *Sports Med*.	Retrospective	23.29	28	18	36.4
			24.88	31	15	41.5
			21.36	15	9	35.8
Tran et al., 2013 [296]	*ANZ J*. *Surg*.	Retrospective	14	34	5	15.7
Ukwuani et al., 2018 [297]	*Arthroscopy*	Retrospective	23	21	62	19.9
				43		23.3
Vahedi et al., 2019 [298]	*J*. *Arthroplasty*	Retrospective	51.6	73	35	30.7
			49.2	550	268	34.5
Vahedi et al., 2019 [299]	*J*. *Arthroplasty*	Retrospective	57.6	51	33	27.4
			49.2	550	253	34.5
Vahedi et al., 2021 [300]	*JB JS Open Access*	Prospective	89.1	156	55	30.8
			87.7	22	10	41.0
Vashneya et al., 2022 [301]	*Arthroscopy*	Retrospective	12	2564	1969	36.7
				11,245	6882	36.2
Wang et al., 2011 [302]	*Orthop*. *Surg*.	Retrospective	11.6	21	12	37.1
Wang et al., 2023 [303]	*Orthop*. *J*. *Sports Med*.	Prospective	112.8	32	24	27.7
Weber et al., 2020 [81]	*Orthop*. *J*. *Sports Med*.	Retrospective		1	2	19.5
				15	2	
				8	1	
				6		
				9	6	
White et al., 2020 [304]	*Arthroscopy*	Retrospective	45.6	136	130	48.1
			67.2	82	76	47.0
			43.2	94	87	34.6
White et al., 2022 [305]	*Arthroscopy*	Prospective	46.2	47	36	42.2
Winge et al., 2021 [306]	*J*. *Hip*. *Preserv*. *Surg*.	Retrospective	80.4	29	18	16.3
		
Wirries et al., 2021 [307]	*Int*. *Orthop*.	Retrospective	51.6	47		43.7
				32		46.2
Wu et al., 2019 [308]	*J*. *Orthop*. *Surg*. *Res*.	Retrospective	44	30	10	40.7
				9	7	53.2
Wyatt et al., 2019 [309]	*Hip*. *Int*.	Retrospective	40.8	27	31	26.7
Yacovelli et al., 2021 [310]	*Clin*. *Orthop*. *Relat*. *Res*.	Retrospective	60	130	70	47.0
				260	133	28.0
Yang et al., 2022 [311]	*Orthop*. *J*. *Sports Med*.	Retrospective	38.4	80	49	36.5
			38.7	80	49	35.4
			37.5	81	49	35.6
			38.5	81	51	37.7
			38.6	80	49	37.6
			38.2	80	50	37.1
Yang et al., 2023 [312]	*Orthop*. *Surg*.	Retrospective	38.2	57	27	53.2
Yang et al., 2023 [313]	*Orthop*. *Surg*.	Retrospective	40	71	22	34.6
			39.6	71	24	35.2
Yin et al., 2021 [314]	*Orthop*. *Surg*.	Retrospective	16	26	14	38.2
			18	30	17	40.1
You et al., 2020 [315]	*Orthop*. *J*. *Sports Med*.	Prospective	24	168	91	35.3
Youngman et al., 2021 [316]	*J*. *Pediatr*. *Orthop*.	Retrospective	37	86	64	16.5
		
Zacharias et al., 2024 [317]	*Arthrosc*. *Sports Med*. *Rehabil*.	Arthroscopy	12	21	13	35.6
				79	54	34.2
Zhang et al., 2021 [318]	*Arthroscopy*	Retrospective	24	16	6	32.4
				11	4	34.0
Zimmerer et al., 2020 [319]	*Orthop*. *J*. *Sports Med*.	Retrospective	43.8	8	24	35.9
				7		
				6		
				15		
Zimmerer et al., 2021 [320]	*Arthroscopy*	Retrospective	132.7	51	21	43.0
			131.2	61	20	44.1
Zimmerer et al., 2021 [321]	*Orthop*. *J*. *Sports Med*.	Prospective	132	44	17	42.2
